# Predict Defibrillation Outcome Using Stepping Increment of Poincare Plot for Out-of-Hospital Ventricular Fibrillation Cardiac Arrest

**DOI:** 10.1155/2015/493472

**Published:** 2015-09-02

**Authors:** Yushun Gong, Yubao Lu, Lei Zhang, Hehua Zhang, Yongqin Li

**Affiliations:** ^1^School of Biomedical Engineering, Third Military Medical University, Chongqing 400038, China; ^2^Emergency Department, Xinqiao Hospital, Third Military Medical University, Chongqing 400037, China; ^3^Emergency Department, Southwest Hospital, Third Military Medical University, Chongqing 400038, China; ^4^Department of Medical Engineering, Daping Hospital and Research Institute of Surgery, Third Military Medical University, Chongqing 400042, China

## Abstract

Early cardiopulmonary resuscitation together with early defibrillation is a key point in the chain of survival for cardiac arrest. Optimizing the timing of defibrillation by predicting the possibility of successful electric shock can guide treatments between defibrillation and cardiopulmonary resuscitation and improve the rate of restoration of spontaneous circulation. Numerous methods have been proposed for predicting defibrillation success based on quantification of the ventricular fibrillation waveform during past decades. To date, however, no analytical technique has been widely accepted for clinical application. In the present study, we investigate whether median stepping increment that is calculated from the Euclidean distance of consecutive points in Poincare plot could be used to predict the likelihood of successful defibrillation. Electrocardiographic recordings of out-of-hospital cardiac arrest patients were obtained from the external defibrillators. The performance of the proposed method was evaluated by receiver operating characteristic curve and compared with the results of other established features. The results indicated that median stepping increment has comparable performance to the established methods in predicting the likelihood of successful defibrillation.

## 1. Introduction

Ventricular fibrillation (VF), which is characterized as rapid and disorganized contractions of the heart with complex electrocardiogram (ECG) patterns, is the most frequent initial rhythm in witnessed out-of-hospital cardiac arrest (OHCA) [1]. Electrical defibrillation, which consists of delivering a therapeutic dose of electrical current to the fibrillating heart with the aid of a defibrillator, is still the only effective way to treat the life-threatening VF. Early cardiopulmonary resuscitation (CPR) together with early defibrillation is a key point in the chain of survival for OHCA patients [2]. The probability of defibrillation success is inversely proportional to the duration of VF. Epidemiological data indicated that, for every minute that passes between collapse and defibrillation, survival rates from witnessed VF decrease by 7%–10% if no CPR is provided. With effective CPR, the success rate of rescue decreases by 3-4% per minute [3]. Clinical studies revealed that defibrillation immediately after the onset of VF usually resulted in restoration of spontaneous circulation (ROSC). When the duration of untreated VF exceeded 4-5 minutes, initial CPR with chest compression before delivery of a defibrillation attempt improved the likelihood of restoring an organized cardiac electrical activity with pulses [4]. However, whether delivering a longer period of CPR before defibrillation improves outcomes in cardiac arrest is still controversial [3–6]. Since delays in and interruptions of CPR degenerate VF into a finer and lower amplitude signal while repeated futile shocks increase the severity of myocardial damage, the ability to gain information concerning the state of the myocardium and to optimize the timing of defibrillation would allow therapy to be tailored to an individual heart [7–11].

ECG waveform, which is routinely available in the current automated external defibrillators (AEDs), has been extensively investigated for the purpose of predicting the probability of defibrillation outcome [12]. The prediction of successful defibrillation allows adjustment of the resuscitation protocol to the condition of the patient, rather than using fixed time intervals in universally applied treatment protocols. Presumably, the organization of the surface ECG has some relationship to the underlying organization of the myocardial electrical activity. During past decades, multiple methods have been proposed for predicting defibrillation success based on VF waveform analysis [13–21]. Approaches for optimizing timing of defibrillation include measures based on time domain methods, frequency domain methods, nonlinear dynamics methods, and a combination of these methods. To date, however, no analytical technique has been widely accepted for clinical application [22].

In the present study, we investigated whether median stepping increment (MSI), a nonlinear method that is calculated by the Euclidean distance of consecutive points in Poincare plot of VF waveform, could predict the success of defibrillation. We also compared the performance of MSI with other established amplitude and frequency based algorithms in OHCA patients who experienced cardiac arrest and CPR.

## 2. Materials and Methods

### 2.1. Data Collection

Data were collected in emergency departments of Southwest hospital, Xingqiao hospital, and Daping hospital in Chongqing, China, between Jan 2012 and Feb 2013. Ethical approval for this retrospective review study was obtained in each hospital. A total of 159 OHCA nontraumatic adult patients with VF/VT as the first recorded rhythm were included in this study. Among the 159 patients, 127 were treated with Philips defibrillator (Philips MRx, Philips Medical Systems, Seattle, WA, USA) and 32 patients were treated with ZOLL defibrillator (M-Series, Zoll Medical Corporation, Chelmsford, MA, USA).

The data was analyzed offline through user-designed software based on Matlab (The MathWorks, Inc., Natick, MA, USA). The sample rate was 250 Hz for ZOLL defibrillators and 200 Hz for Philips defibrillators. ECG waveforms recorded by Philips defibrillators were digitally resampled to 250 Hz for compatibility. An episode of 2.05 seconds (512 data points) ending at 0.5 seconds before the first shock attempt on each patient was selected for analysis. For preprocessing purpose, 4th order Butterworth band-pass filter (3–45 Hz) was used to remove ECG baseline drifting, compression artifacts, and electrical power interference.

The preshock and postshock rhythms were annotated by two cardiologists who were blinded to waveform analysis. VF was defined as a disordered electrical activity without the presence of observable QRS and with a peak-to-peak voltage greater than 0.1 mV. Ventricular tachycardia (VT) was defined as resting heart rate > 150 beats/min. A defibrillation was regarded as successful when VF/VT was converted to an organized rhythm with heart rate ⩾ 40 beats/min within one minute after each defibrillation [23]. And unsuccessful defibrillation was confirmed if VF/VT, asystole, or pulseless electrical activity occurred [24].

### 2.2. Stepping Increment of the Poincare Plot

**Figure 1:**
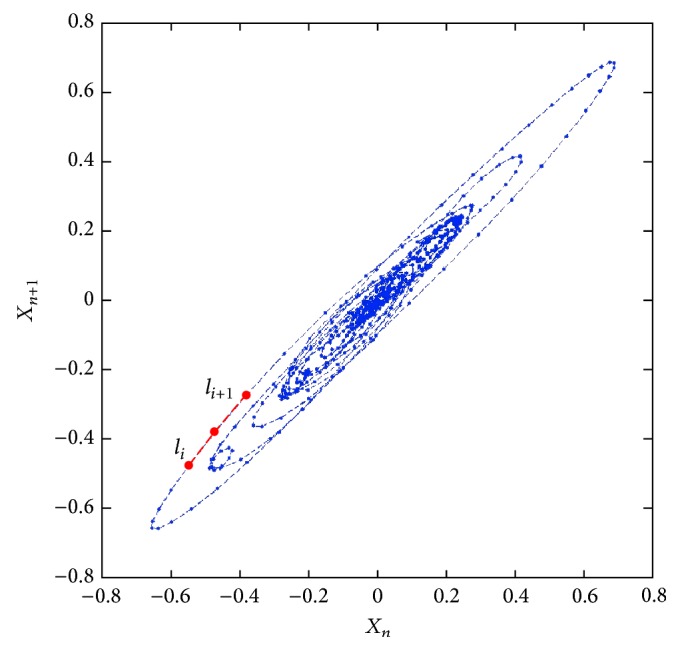
Example of Poincare plot of VF waveform and the stepping increment of the constructed two-dimensional diagrams.

**Figure 2:**
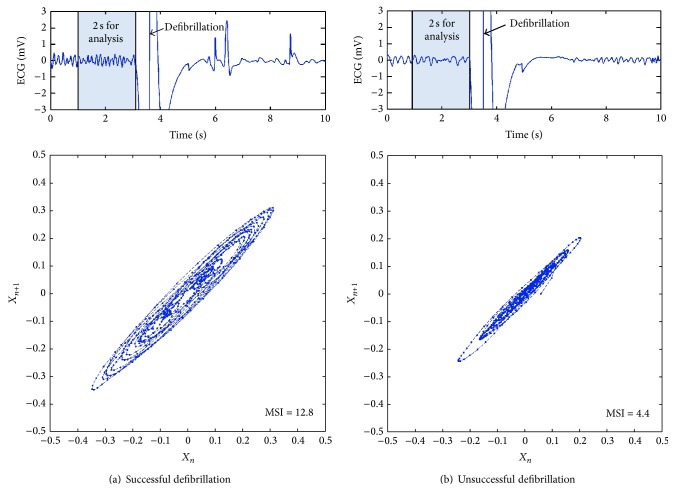
Examples of successful and unsuccessful shocks. (a) VF waveform and Poincare plot for a successful shock. (b) VF waveform and Poincare plot for an unsuccessful shock.

Poincare plot analysis provides a visual tool to characterize the complex feature of time series fluctuations and measures the self-similarity in processes [25]. To create a Poincare plot, the VF waveform *S* = [*x*_0_, *x*_1_, *x*_2_,…, *x*_*n*−1_] is plotted on the *x*-axis and *S*′ = [*x*_1_, *x*_2_, *x*_3_,…, *x*_*n*_] on the *y*-axis to form a two-dimensional diagram (Figure 1). Stepping increment *l*_*i*_ is defined as the Euclidean distance of consecutive points in the Poincare plot of VF waveform [26]:

(1) $$\begin{array}{c} l_{i}=\left(\sqrt{{\left(x_{i+1}-x_{i}\right)}^{2}+{\left(x_{i+2}-x_{i+1}\right)}^{2}}\right)\cdot f_{s}, \end{array}$$

where *x*_*i*_ represents the magnitude at the *i*th sample and *f*_*s*_ is the sampling rate. The median length of the stepping increment (MSI) = median(*l*_*i*_) is used as the measure to predict the probability of successful defibrillation.  Figure 2 shows an example of successful and unsuccessful prediction of defibrillation using the proposed method.

### 2.3. Comparative Methods

Three established measures, including amplitude spectrum area (AMSA), signal integral (SignInt), and mean slope (MS), were also calculated and compared with the proposed method because of their relatively better performance compared with other measures in earlier clinical studies [13–17].

AMSA describes the amplitude-weighted mean frequency [13–15] and is calculated as the summed product of frequency (*F*_*i*_) and amplitude (*A*_*i*_) over an interval of 4 to 48 Hz of the Fourier transformed signal:

(2) $$\begin{array}{c} AMSA=\frac{1}{N}\overset{48}{\underset{i=4}{\sum }}A_{i}\cdot F_{i}. \end{array}$$

SignInt measures the amplitude of the signal [16] and is calculated by the sum of the absolute amplitude in a certain time interval:

(3) $$\begin{array}{c} SignInt=\left|x_{1}\right|+\left|x_{2}\right|+\cdots +\left|x_{i}\right|+\cdots +\left|x_{n}\right|. \end{array}$$

MS measures the overall slope of the selected VF segment [13] and is calculated by the average of the slope between two points:

(4) $$\begin{array}{c} MS=\frac{1}{N}\overset{N}{\underset{i=1}{\sum }}\left|x_{i}-x_{i-1}\right|\cdot f_{s}, \end{array}$$

where *x*_*i*_ represents the magnitude at the *i*th sample, *f*_*s*_ is the sampling rate, and *N* is the length of signal.

### 2.4. Statistics

All the data were expressed as mean ± SD. Comparisons were conducted between successful and unsuccessful first defibrillation by using the independent samples *t*-test. A receiver operating characteristic (ROC) curve was constructed to show the efficiency of each prediction method. The discriminative ability of the waveform parameters was evaluated by determining the area under ROC curve (AUC) and its 95% confidence interval (CI). Correlations among pairs of different VF waveform measures were evaluated by determination of Pearson correlation coefficients. A *p* value of 0.05 was considered as statistically significant.

## 3. Results

**Figure 3:**
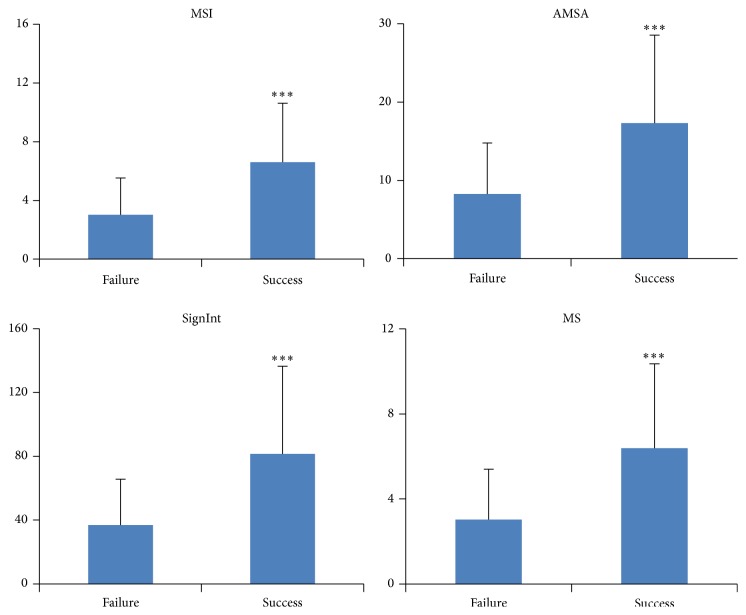
Measures between successful shocks (success) and unsuccessful shocks (failure). Median stepping increment of Poincare plot, MSI; amplitude spectrum area, AMSA; signal integral, SignInt; mean slope, MS; *∗∗∗*, compared with unsuccessful shocks, *p* < 0.001.

Among 159 first defibrillation shocks, 38.4% were annotated as successful and 61.6% were annotated as unsuccessful according to the adopted definition.  Figure 3 shows the value of different measures between successful and unsuccessful shocks. Compared with unsuccessful shocks, the successful ones had significant higher values of MSI (6.3 ± 3.2 versus 2.9 ± 2.4, *p* < 0.001), AMSA (18.2 ± 11.9 versus 7.9 ± 6.3, *p* < 0.001), SignInt (78.2 ± 45.0 versus 36.8 ± 28.9, *p* < 0.001), and MS (6.3 ± 3.6 versus 3.0 ± 2.3, *p* < 0.001).

**Figure 4:**
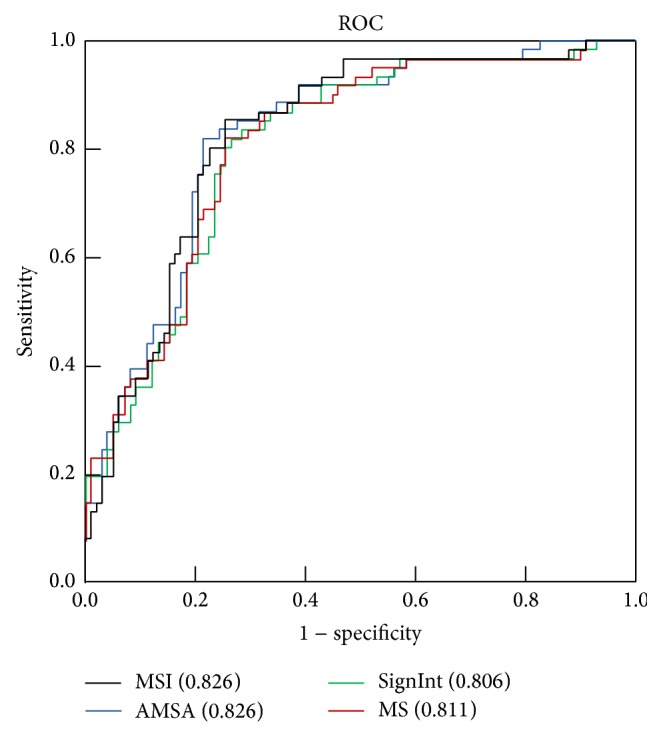
Receiver operating characteristic (ROC) curve for the prediction of defibrillation outcome using different measures.

Figure 4 shows the ROC curves for the prediction of successful defibrillation with MSI, AMSA, MS, and SignInt. The AUC was 0.826 (95% CI = 0.760–0.891) for MSI, 0.826 (95% CI = 0.760–0.891) for AMSA, 0.806 (95% CI = 0.737–0.875) for SignInt, and 0.811 (95% CI = 0.743–0.879) for MS. No statistical differences in AUC were observed among the compared methods.

The Pearson correlation coefficient was 0.873 between MSI and AMSA, 0.931 between MSI and SignInt, 0.946 between MSI and MS, 0.875 between AMSA and SignInt, 0.945 between AMSA and MS, and 0.958 between SignInt and MS (*p* < 0.001 for all pairs).

## 4. Discussion

The results of this study suggest that a nonlinear VF waveform analysis method, that is, MSI, can predict successful defibrillation in OHCA patients as efficiently as other established methods using amplitude and frequency based analysis.

The search for a reliable predictor of successful defibrillation obtained from the analysis of VF began almost 30 years ago after the observation that the success rate of defibrillation was associated with the time elapsed following VF onset and the performance of CPR; both of the aforementioned variables can alter the electrical features of the VF waveforms [27–29]. Measures of the VF waveform enable better allocation of cardiac arrest treatment by determining which patients should receive immediate defibrillation rather than CPR. Although numerous algorithms have been developed and these measures provided encouraging results for optimizing the timing of defibrillation in both animal and clinical studies, there are still concerns that limit their implementation through clinical devices. For the methods using amplitude information, the recording conditions, movement artifact, recording devices, body habitus, electrode placement, and transthoracic impedance may alter measured VF features [30, 31]. Even though frequency analysis to assess the VF waveform overcomes some of the problems encountered with amplitude analysis, the technique is suitable only for analysis of stationary signals where the waveform does not change. Given the physiologic deterioration in the myocardium during cardiac arrest, this assumption may not apply in VF.

Earlier studies demonstrated that VF waveform was a nonstationary, complex but nonrandom process [32, 33], which could not be accurately characterized by statistical properties. Therefore, VF features based on amplitude and frequency analysis might miss information of the nonlinear characteristics in VF waveform and lead to an incorrect decision. Methods based on nonlinear analysis were then introduced to characterize VF waveforms [19–21]. Compared with amplitude and frequency based methods, the fractal self-similarity dimension provides a quantitative description of the nonlinear characteristics of VF waveform morphology. In an earlier study, Callaway et al. [19] showed that the scaling exponent (ScE) of VF waveform based on fractal analysis could predict the probability of defibrillation success for first shocks. Menegazzi et al. [20] confirmed that ScE could be used to guide the timing of defibrillation. Sherman et al. [34] derived a new measure based on the roughness of the VF waveform and the Logarithm of the Absolute Correlations (LAC) and compared it with ScE for the prediction of duration of VF and the likelihood of ROSC. Their results showed that AUC at a high sample rate was similar for LAC compared to ScE but greater for LAC compared to ScE at a low sample rate. Recently, Lin et al. [21] predicted defibrillation success by detrended fluctuation analysis (DFA), a statistical self-affinity of the VF waveform. Their results showed that VF waveform analysis based on amplitude-independent DFA could help predict first shock defibrillation success in patients with OHCA. However, the reported AUC of 0.63 was relatively small.

In the present study, a nonlinear index was shown to be helpful in optimizing the timing of defibrillation. Unlike ScE which was focused on the fractal dimension and complexity, MSI was calculated through the Euclidean distance of consecutive points in Poincare plot. The nonlinear analysis method of Poincare plot could measure the feature of signal fluctuations and the self-similarity in processes [25], which is suitable for VF waveform analysis. In the proposed method, the Euclidean distance between two points in the Poincare plot contains the information of two consecutive amplitude changes of the original signal. It reflects the amplitude and frequency features which are similar to those in AMSA, SignInt, and MS; therefore, tight correlations were observed among these measures. However, the adjacent features of the sampling points are grouped by MSI and may offer additional information on the local trend and self-similarity of the VF waveform. Clinical data showed that MSI was significantly higher in the successful shock and confirmed that MSI could quantify the organization of VF and thus predict defibrillation outcomes. Compared with the established methods SignInt and MS, the AUC was slightly higher, even though no statistical difference was obtained. Furthermore, the nonlinear analysis based on an MSI corresponding to a 2-second-lasting VF waveform enables prompt and reliable prediction of successful defibrillation.

Our study might have some limitations. Firstly, the ECG episodes were selected from artifact-free signals immediately before defibrillation. Therefore, the effects of CPR related artifacts on the performance of the proposed method are unknown. Secondly, the proposed method was only shown to be helpful in predicting successful defibrillation as defined in the current study, but the association between MSI and ROSC or long term survival has not been investigated. Thirdly, the studied sample size of OHCA patients was relatively small, and further work will be required to validate the clinical usefulness of the current method.

## 5. Conclusion

The results of this study suggest that a nonlinear measure, that is, MSI calculated from 2 seconds of ECG waveform, may predict the likelihood of successful defibrillation promptly and reliably in patients with OHCA.
